# The Influence of Aerobic and Strength Training on Oxidative Stress and Antioxidant Status in Hemodialysis Patients: A Randomized Controlled Trial

**DOI:** 10.1002/hsr2.72058

**Published:** 2026-03-09

**Authors:** Yuwei Chen, Lu Yin, Xinzhou Zhang, Xue Zheng, Lijuan Lan, Liping Sun

**Affiliations:** ^1^ Department of Nephrology, Shenzhen People's Hospital, The Second Clinical Medical College Jinan University Shenzhen China; ^2^ Department of Nephrology, Shenzhen Key Laboratory of Renal, Shenzhen People's Hospital (The Second Clinical Medical College Jinan University; The First Affiliated Hospital, Southern University of Science and Technology) Shenzhen Guangdong China

**Keywords:** body composition, chronic renal disease, exercise, infammation, maintenance hemodialysis, oxidative stress

## Abstract

**Background and Aims:**

To investigate the effects of a 6‐month supervised, personalized combined aerobic and resistance training program on oxidative stress, antioxidant capacity, infection‐related biomarkers, and body composition in maintenance hemodialysis (MHD) patients.

**Methods:**

In this single‐center, randomized controlled trial (nonblinded, parallel design), 61 MHD patients (standard thrice‐weekly 4‐h sessions) were randomly assigned to either an exercise group (*n* = 36) or a control group (*n* = 35). The exercise intervention comprised: The exercise group received 6 months of supervised training (three times a week, 2 h before dialysis), including: ①Aerobic exercise: A 20‐min stationary cycling session at RPE 13 (with heart rate maintained at 60%–70% of maximum heart rate). ②Resistance training: Four lower‐body exercises (leg extension, straight leg raise, hip abduction, hip flexion) using ethylene‐vinyl acetate resistance bands (10 reps/set, RPE 13). The control group received standard care. Primary outcomes were oxidative stress markers (MDA, AOPP), antioxidant indicators (CAT, THIOL, TBIL), infection markers (IL‑6, PCT, CRP, Hcy), and body composition measures (muscle mass, BMI).

**Results:**

After 6 months of intervention, the exercise group exhibited significantly reduced levels of MDA (*p* < 0.05) and AOPP (*p* < 0.05). CAT activity (*p* < 0.05), THIOL levels (*p* < 0.05), and TBIL levels (*p* < 0.001) were significantly increased. IL‐6, PCT, CRP, and Hcy were lower than those in the control group (*p* < 0.05). The exercise group also demonstrated improved Kt/V (*p* < 0.05) and attenuated declines in muscle mass and BMI compared to controls (*p* < 0.05).

**Conclusion:**

This structured exercise program effectively mitigates oxidative damage, boosts antioxidant defenses, reduces infection markers, and enhances dialysis efficiency and body composition in MHD patients. These findings provide evidence for incorporating supervised exercise as a novel therapeutic strategy in nephrology care.

## 1 Introduction

Chronic kidney disease (CKD) burden is rising globally, with maintenance hemodialysis (MHD)—primary renal replacement therapy for end‐stage CKD—seeing significant recent increases in prevalence [1]. MHD patients inevitably develop multiple impairments, with physical dysfunction as a key consequence, restricting daily activities and linking to poor outcomes (higher mortality, accelerated cardiovascular disease, increased infection risk) [2, 3, 4]. Targeted rehabilitation for physical dysfunction is thus an urgent unmet need.

Beyond kidney damage, MHD patients face chronic systemic oxidative stress (OS), driven by excessive reactive oxygen species (ROS) and impaired antioxidant defenses. Contributing factors include uremic toxins [e.g., advanced glycation end products, homocysteine], dialysis‐specific issues (e.g., bioincompatible membranes, cytokine activation), and metabolic derangements [e.g., iron overload, reduced SOD/CAT activity (superoxide dismutase/catalase activity)] [5, 6]. Hemodialysis itself exacerbates OS via blood‐membrane interactions activating neutrophils, creating a vicious cycle of oxidative‐inflammation crosstalk that promotes endothelial dysfunction, chronic inflammation, and atherosclerosis (the leading mortality cause in MHD) [7].

Exercise is a cornerstone of MHD rehabilitation [8, 9]. It enhances muscle function via myogenesis, regeneration, and myostatin modulation [10]. However, MHD patients have reduced exercise tolerance (due to sarcopenia, malnutrition, acidosis, and inactivity). Combined aerobic‐resistance training (CTRT) outperforms single‐mode interventions in improving cardiorespiratory fitness, muscle strength, and function [11].

While exercise benefits physical function, its dual role in OS remains debated: acute exercise may transiently increase ROS (via mitochondrial/neutrophil activation) [12], but long‐term regular exercise upregulates antioxidants [SOD, CAT, GPx (glutathione peroxidase)] to restore redox balance. Yet, existing studies focus on isolated aerobic/resistance training, with limited data on supervised, personalized CTRT, the most clinically relevant modality for MHD. Additionally, the impacts of acute/chronic exercise on key oxidative biomarkers [MDA, myeloperoxidase] and inflammatory mediators (interleukin‐6 [IL‐6], C‐reactive protein [CRP], procalcitonin [PCT]) in MHD are inconclusive (e.g., Esgalhado et al. [13] reported acute resistance exercise reduced SOD without altering CAT/GPx, highlighting modality‐specific effects).

Infection complications are further challenging: chronic inflammation/OS impair immune function (neutrophil/lymphocyte dysfunction), increasing vulnerability to infections (a major cause of hospitalization/mortality) [14]. However, the interplay between exercise, OS, and infection biomarkers (IL‐6, PCT, CRP, homocysteine [Hcy]) in MHD is understudied.

This RCT investigates a 6‐month supervised, personalized CTRT program's effects on OS [MDA, AOPP (advanced oxidation protein products)], antioxidant capacity [CAT, THIOL (Thiol), TBIL (total bilirubin)], infection/inflammation markers (IL‐6, PCT, CRP, Hcy), and clinical outcomes (dialysis efficacy, body composition) in MHD patients, aiming to optimize exercise‐based rehabilitation strategies.

## 2 Materials and Methods

### 2.1 General Information and Study Design

This nonblinded, randomized, parallel‐controlled trial investigated supervised exercise effects in MHD patients. Participants were recruited from Shenzhen People's Hospital Hemodialysis Center (April 2024–March 2025), requiring regular hemodialysis (3 × /week, 4 h/session) for ≥ 6 months preenrollment.

Inclusion Criteria: (1) Age < 70 years; (2) Dialysis ≥ 6 months with stable condition; (3) BP 100–160/60–100 mmHg; (4) Autologous arteriovenous fistula as vascular access; (5) Written informed consent provided.

Exclusion Criteria: Severe osteoarticular disorders (walking < 200 m); uncontrolled cardiovascular disease ([e.g., heart failure, unstable angina]; psychoactive substance use history; stroke/hospitalization in past 6 months; cognitive impairment (unable to cooperate with exercise assessments); acute/chronic conditions posing exercise safety risks (e.g., uncontrolled DM complications, severe anemia).

#### 2.1.1 Randomization and Group Allocation

**Figure 1:**
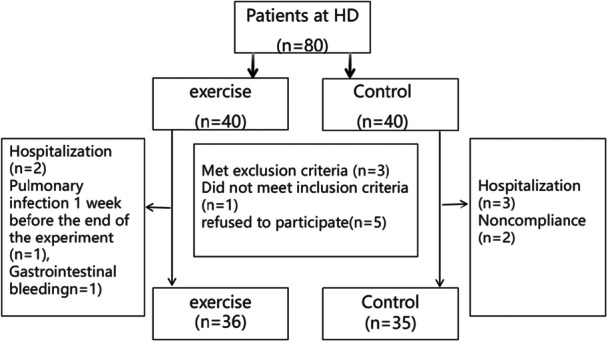
Flowchart of the participants.

Eighty eligible patients were randomized (1:1) to exercise (*n* = 40) or control (*n* = 40) groups via computer‐generated random numbers, with balanced baselines. During the study, 4 (10.0%) exercise participants withdrew (personal reasons), and 5 (12.5%) control participants withdrew (transfer/poor adherence). These withdrawn patients were excluded from the final analysis, leaving 71 for final analysis (exercise: *n* = 36; control: *n* = 35; Figure 1). All adhered to the protocol with standardized instructions. In the evaluation of efficacy measures, this study included all randomized subjects in the final statistical analysis and strictly classified them according to their initial randomization groups. The study was carried out after the protocol was approved by the Ethics Committee of Shenzhen People's Hospital (approval number LL‐KY‐ 2025147‐02), and written informed consent was obtained from all patients before commencement of the study.

#### 2.1.2 Blinding and Intervention Delivery

Due to dialysis unit space constraints, groups shared the treatment area, precluding patient/staff blinding (nonblinded design). The exercise intervention was tailored by a certified physical therapist (based on individual function) and supervised by a consistent hemodialysis specialist nurse, who oversaw all sessions and outcome evaluations for consistency. Controls received standard dialysis care (no exercise guidance).

### 2.2 Research Design and Patient Management

#### 2.2.1 Exercise Intervention Protocol

The exercise group underwent a 6‐month, supervised, individualized, combined aerobic, and resistance training program administered three times weekly. Sessions were consistently scheduled 2 h before each hemodialysis session to align with clinical workflows and minimize disruption to dialysis efficacy. The intervention was structured into two complementary modalities:

##### 2.2.1.1 Aerobic Training

Participants completed 20 min of continuous cycling using an electronically braked stationary bike (Terasu Ergo II; Showa Denki, Osaka, Japan). Exercise intensity was rigorously monitored via dual metrics to ensure precision, particularly critical for patients on beta‐blockers (where heart rate [HR] reliability may be compromised): A target zone of 60%–70% of maximum HR (calculated as 220—age) was set, with real‐time HR tracked using a chest strap monitor (Polar H10; Polar Electro, Kempele, Finland). A target RPE of 13 (slightly tired, slightly sweaty, without palpitations or shortness of breath) was maintained, with verbal feedback provided by trained instructors to adjust intensity dynamically. Dual monitoring ensured intensity precision, particularly for patients on beta‐blockers (where HR reliability may be compromised).

##### 2.2.1.2 Resistance Training

Four lower‐limb resistance exercises were performed using ethylene‐vinyl acetate resistance bands (Guangdong, China), selected for their portability, safety, and adaptability to varying fitness levels: Seated with the band anchored at the ankle, participants extended one leg forward against resistance; Supine with the band looped around the foot, participants lifted one leg to a 45° angle while keeping the knee straight; Standing with the band anchored at the ankle, participants abducted one leg outward against resistance; Seated with the band anchored at the ankle, participants lifted one knee toward the chest against resistance. Each exercise was structured as follows: 10 repetitions per set, with 2–3 sets per exercise (progressively adjusted based on tolerance); Each repetition included 5 s of isometric contraction (muscle tension without joint movement) followed by 5 s of eccentric contraction (controlled lengthening under tension), emphasizing muscle endurance and strength; Target intensity was aligned with the aerobic HR zone (60%–70% max HR) and maintained at RPE 13 to ensure coherence between aerobic and resistance components.

##### 2.2.1.3 Training Procedure

Session structure: Each 2‐h predialysis session began with a 5‐min supine stretching warm‐up (to prevent injury and optimize vascular access accessibility). All exercises were conducted in the supine position (standard for predialysis safety and vascular access preservation).

Environmental Control: Throughout the intervention period, dialysis parameters (blood flow rate: 100–350 mL/min; dialysate flow rate: 500 mL/min), medication regimens (including statins, antioxidants, and vitamins), and lifestyle factors (dietary intake, sleep patterns) were strictly maintained at baseline levels to isolate the effect of exercise.

##### 2.2.1.4 Safety Monitoring and Contraindications

Pre/postsession assessments: Nurses measured blood pressure (BP), HR, blood oxygen saturation (SpO₂), and interdialytic weight gain (IDWG) before and after each exercise session to detect acute physiological responses. Contraindications for Participation: Patients with any of the following were excluded from the exercise program: BP > 180/110 mmHg or < 110/50 mmHg; HR < 60 bpm; SpO₂ < 88%; IDWG > 5% between dialysis sessions; or inability to establish stable vascular access.

Emergency termination criteria: Exercise was immediately halted if participants reported chest pain, dizziness, or dyspnea, or if nurses observed abnormal vital signs (e.g., HR/BP exceeding safe thresholds).

#### 2.2.2 Blood Sample Collection and Laboratory Analysis

To reduce confounding variables, strict standardized protocols were followed. Participants avoided strenuous activity 24 h presampling. Samples were collected at a fixed morning time (07:00–07:30 a.m.) to control diurnal variability, prior to dialysis. Blood was drawn sterilely from the median cubital vein (10 mL total) into three tubes: EDTA (3.5 mL, whole blood analysis, e.g., CBC); lithium heparin (4 mL, plasma assays, e.g., OS markers); SST (2.5 mL, serum biomarkers, e.g., TBIL, hormones). Serum was separated by centrifugation (2500 × g, 10 min, 4°C), then plasma/serum aliquots stored at –80°C long‐term. All assays used standardized reagents in a single centralized lab to minimize variability; preanalytical conditions (fasting, timing, centrifugation) were strictly enforced, with samples checked for hemolysis/lipemia before freezing.

#### 2.2.3 CAT Activity Assay

CAT enzymatic activity was quantified via spectrophotometric measurement of H₂O₂ decomposition rate at 240 nm wavelength (JENWAY 6105 UV‐Vis spectrophotometer) at 25°C under standardized conditions.

#### 2.2.4 Body Composition Analysis

Skeletal muscle mass, body fat mass, body mass index (BMI), and visceral fat area were assessed using bioelectrical impedance analysis (BIA) (IOI 353, Jawon, Korea) with the following protocols: measurements conducted in the morning (10:00–12:00 a.m.) after a 3‐h fast to minimize postprandial fluid shifts. Participants were instructed to void bladder/bowel and stabilize for 10 min in an upright position (to ensure hydration equilibrium). Impedance values (ohms), height, and weight were input into the device's validated proprietary algorithm to estimate compartmental body composition.

#### 2.2.5 Statistical Analysis

Analyses were performed using SPSS 26.0 (IBM Corp, Armonk, NY, USA). Normality was assessed via Shapiro–Wilk (*n* ≤ 50) or Kolmogorov–Smirnov (*n* > 50) tests, with histogram validation. Normal data (confirmed by tests/histograms): mean ± SD (x̄ ± SD); between‐group (exercise vs. control) and within‐group (pre vs. postintervention) comparisons at baseline/6 months used independent/paired *t*‐tests, respectively. Nonnormal data (failed tests/skewed): median [IQR, M(P₂₅, P₇₅)]; between‐group comparisons used the Mann–Whitney *U*‐test. Categorical data: %; group comparisons used chi‐square/Fisher's exact test. Body composition was compared between groups using Analysis of Variance (ANOVA). The *F*‐value, the core statistic in ANOVA, indicates whether group mean differences are statistically significant. A higher *F*‐value suggests a clearer effect of the intervention on the outcomes and a lower likelihood that the observed differences are merely due to individual variation among patients. Significance: *p* < 0.05.

## 3 Results

### 3.1 General Information and Baseline Characteristics

A total of 71 MHD patients were included (41 males [57.7%], 30 females [42.3%]; mean age 54.0 ± 9.7 years; median dialysis duration 50.36 months [IQR 25.75–69.37]). No significant baseline differences existed between the exercise (*n* = 36) and control (*n* = 35) groups in demographics, dialysis duration, Kt/V, BP, BMI, and metabolic profiles (Table 1). Primary end‐stage renal disease causes (Table 2): chronic glomerulonephritis (23 cases, 32.4%; 12 vs. 11), diabetic kidney disease (21, 29.6%; 11 vs. 10), hypertensive nephropathy (19, 26.8%; 10 vs. 9), and others (8, 11.3%; 3 vs. 5). Distributions were balanced between groups (*p* = 0.92, chi‐square test).

**Table 1:** Comparison of baseline demographic and clinical characteristics between the two groups (*n*[%], [*x* ± *s*], *M*[*P*_25_, *P*_75_]).

Group	the exercise group (*n* = 36)	the control group (*n* = 35)	statistical value	*p*‐value
Male/female (*n*)	21/15	20/15	*χ*² = 0.067	0.842
Age (years)	51.19 ± 9.13	52.84 ± 8.45	*t* = 0.852	0.416
Dialysis age (months)	51.5 (26.3, 76.0)	47.0 (29.8, 75.0)	*Z* = –0.476	0.623
BMI (kg/m²)	23.69 ± 2.10	23.61 ± 2.04	*t* = –0.156	0.861
Mean arterial pressure (mmHg)	107.83 ± 10.37	108.24 ± 9.69	*t* = 0.164	0.865
Hemoglobin (g/L)	94.76 ± 13.53	93.6 ± 12.16	*t* = –0.407	0.680
Serum albumin (g/L)	38.52 ± 3.19	40.03 ± 3.74	*t* = 1.826	0.059
Blood potassium (mmol/L)	5.34 ± 0.93	5.28 ± 0.77	*t* = 0.917	0.362
Blood calcium (mmol/L)	2.03 ± 0.21	2.09 ± 0.22	*t* = –0.920	0.354
Blood phosphorus (mmol/L)	1.89 ± 0.36	1.95 ± 0.38	*t* = 0.725	0.462
Blood parathyroid hormone (pg/mL)	315.13 ± 105.19	299.89 ± 128.42	*t* = –0.545	0.573
Kt/V	1.26 ± 0.09	1.27 ± 0.08	*t* = 0.446	0.646

Abbreviations: BMI, body mass index; Kt/V, urea removal index.

**Table 2:** Comparison of the composition of primary diseases between the two groups (*n*[%]).

Group	The exercise group (*n* = 36)	The control group (*n* = 35)	Statistical value	*p*‐value
Chronic glomerulonephritis	12 (33.3)	11 (34.3)	*χ*² = 0.052	0.820
Diabetic kidney disease	11 (30.6)	10 (28.6)	*χ*² = 0.060	0.828
Hypertensive nephropathy	10 (27.8)	9 (25.7)	*χ*² = 0.302	0.583
Others	4 (11.1)	4 (11.4)	*χ*² = 0.371	0.556

### 3.2 Changes in Oxidative Damage Markers

After 6 months of supervised exercise, the exercise group (*n* = 36) showed significant reductions in oxidative damage markers (MDA and AOPP) compared to both baseline and the control group (*n* = 35), while the control group exhibited no significant changes (Table 3) Exercise group MDA levels decreased significantly versus controls (*t* = 2.281, *p* = 0.028; Table 3) and versus baseline (*p* < 0.05). In contrast, the control group showed no significant change in MDA levels from pre to postintervention (*t* = 0.293, *p* = 0.771). The exercise group exhibited a marked reduction in AOPP levels after 6 months, with a large effect size (Cohen's *d* = 2.573, *p* = 0.013). No significant difference in AOPP levels was observed in the control group before and after the intervention (*p* > 0.05). These results indicate the 6‐month supervised exercise program effectively attenuates OS in MHD patients.

**Table 3:** Changes in oxidative damage markers in the two groups after 6 months of exercise (x̄ ± *s*).

Group	MDA (umol/L)		AOPP (umol/L)	
	Pro‐exercise	Postexercise	Pro‐exercise	Postexercise
exercise (*n* = 36)	8.09	6.12	62.5	56.2
control (*n* = 35)	8.01	8.07	62.3	63.5
*t*/*d* value	0.293	2.281	0.168	2.573
*p*‐value	0.771	0.028	0.867	0.013

### 3.3 Changes in Antioxidant System Markers

**Figure 2:**
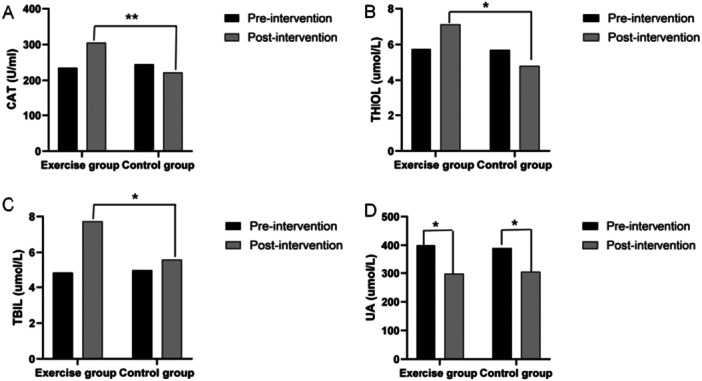
Changes in antioxidant system markers over 6 months of exercise (Data presented as mean ± SD, *p < 0.05, **p < 0.01).

After 6 months of supervised exercise, the exercise group (*n* = 36) showed significant improvements in key antioxidant markers compared to the control group (*n* = 35), with notable within‐group and between‐group differences (Figure 2). Exercise group CAT activity increased significantly from baseline (*p* < 0.05; large effect size, Cohen's *d* = 1.51) and was markedly higher than controls (*p* < 0.01), indicating enhanced enzymatic antioxidant capacity. THIOL (nonenzymatic antioxidant, e.g., glutathione and cysteine) levels rose significantly in the exercise group versus baseline (*p* < 0.05) and controls (*d* = 1.281, *p* = 0.024), reflecting strengthened nonenzymatic defenses. TBIL (endogenous antioxidant) levels increased significantly from baseline (*p* < 0.05) and were higher in the exercise group versus controls (*d* = 1.672, *p* = 0.018), highlighting boosted indirect antioxidant capacity. UA decreased in both groups (*p* < 0.05), likely due to dialysis clearance or metabolic adaptation, with no between‐group difference (*d* = 0.162–0.158, *p* > 0.8), showing no exercise‐specific effect. Overall, the 6‐month exercise program effectively enhanced antioxidant capacity via enzymatic (CAT) and nonenzymatic (THIOL, TBIL) pathways, while UA dynamics remained unaffected.

### 3.4 Changes in Infection‐Related Inflammatory Markers

**Figure 3:**
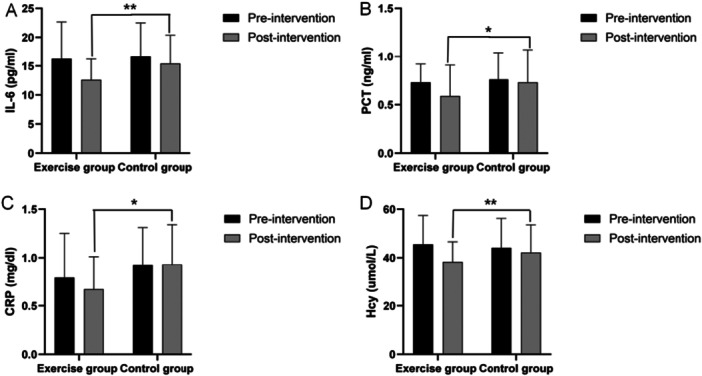
Changes in infection‐related inflammatory markers over 6 months of exercise (mean ± SD).

At baseline, there were no significant differences between the exercise and control groups in serum levels of IL‐6, PCT, CRP, or Hcy (*p* > 0.05). After the intervention, the exercise group exhibited significant reductions in IL‐6 (*t* = 4.368, *p* < 0.001), PCT (*t* = 2.281, *p* = 0.028), CRP (*t* = 2.035, *p* = 0.048), and Hcy (*t* = 5.573, *p* < 0.001). In contrast, the control group showed no significant changes in these markers (*p* > 0.05). Between‐group comparisons revealed that the exercise group had significantly lower postintervention levels of IL‐6 (*p* < 0.01), PCT (*p* < 0.05), CRP (*p* < 0.05), and Hcy (*p* < 0.05) compared to the control group (Figure 3).

### 3.5 Changes in Dialysis Efficiency

**Figure 4:**
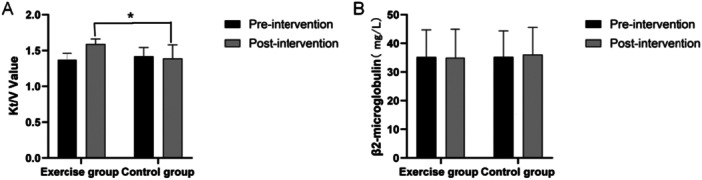
The changes of Kt/V value and Serum β₂‐microglobulin levels (mean ± SD, *p < 0.05). A: Kt/V value; B: Serum β₂‐microglobulin levels.

After 6 months of intervention, the urea clearance index (Kt/V) (Figure 4A) and serum β₂‐microglobulin levels (Figure 4B) showed distinct trends between the exercise and control groups. The exercise group demonstrated a significant improvement in Kt/V (*p* < 0.05), whereas the control group showed no significant change. Between‐group comparison confirmed that the exercise group had a significantly higher Kt/V postintervention, suggesting that exercise may enhance or maintain dialysis efficiency. The exercise group maintained more stable β₂‐microglobulin levels(pre: 35.24 ± 9.45 mg/L, post: 34.98 ± 9.92 mg/L, *p* > 0.05). The control group was 35.28 ± 9.07 mg/L before the intervention and increased to 36.05 ± 9.5 mg/L after the intervention. A comparison between the two groups revealed that the serum β₂‐microglobulin level of the exercise group was relatively more stable after the intervention, while the control group showed a certain degree of accumulation tendency.

### 3.6 Changes in Body Composition

**Figure 5:**
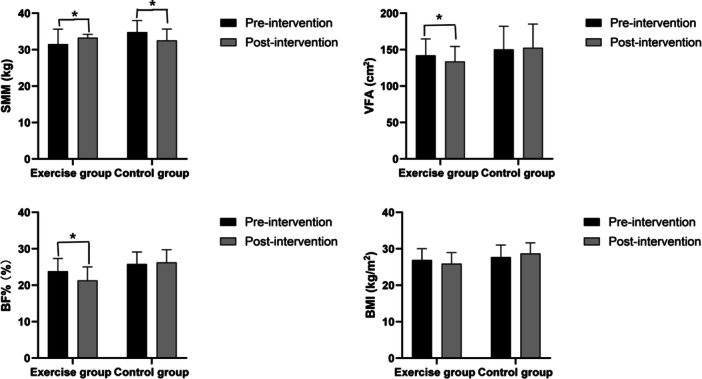
Comparison of BMI, VFA, BF% and SMM between the intervention group and the control group (*p < 0.05).

Over the 6‐month period, the control group exhibited significant unfavorable changes in body composition (Figure 5). In the control group, the whole‐body skeletal muscle mass (SMM) (*F* = 2.249, *p* < 0.05) decreased over time, while the BMI (*F* = 1.512, *p* < 0.05), visceral fat area (VFA) (*F* = 2.012, *p* < 0.05), and whole‐body fat ratio (BF) (*F* = 2.051, *p* < 0.05) increased over time. In contrast, the exercise group showed significant beneficial changes, the muscle mass (*F* = 3.076, *p* < 0.01) was significantly higher after the intervention compared to the control group, while the BMI (*F* = 2.128, *p* < 0.05), VFA (*F* = 3.339, *p* < 0.01), and BF (*F* = 2.865, *p* < 0.05) were all reduced compared to preintervention levels.

## 4 Discussion

This study assessed the effects of a 6‐month supervised, personalized combined aerobic and resistance training intervention on OS, antioxidant defense, infection markers, and body composition in MHD patients. Results demonstrated significant improvements in OS status, enhanced antioxidant capacity, suppressed inflammatory responses, and optimized dialysis efficiency and body composition following the intervention, providing robust evidence‐based support for clinical exercise prescription strategies in MHD populations.

### 4.1 Multi‐Target Regulation of OS and Antioxidant Systems by Exercise

OS, a central driver of cardiovascular complications and microinflammation in MHD patients [15], is characterized by excessive ROS production and impaired antioxidant defense. Our study demonstrated that a 6‐month supervised exercise intervention significantly alleviated OS through multi‐target mechanisms.

First, exercise markedly reduced lipid peroxidation (evidenced by lower MDA, a key lipid oxidation marker; *p* < 0.05), aligning with prior research [16], likely via enhanced mitochondrial function and ROS suppression. Notably, AOPP—an oxidative protein damage biomarker—also decreased, confirming exercise's inhibitory effect on protein‐level OS; reduced neutrophil activation/dialysis‐related inflammatory factor release may contribute, though mechanisms require further study.

Beyond damage markers, exercise upregulated antioxidant defenses: CAT activity (*p* < 0.01) and THIOL levels (*p* < 0.01) rose significantly, indicating enhanced H₂O₂ scavenging (via enzyme gene expression) and nonenzymatic reserves (e.g., glutathione). TBIL also increased (*p* < 0.05), possibly linked to exercise‐enhanced bilirubin metabolism/HO‐1 pathway activation—an emerging antioxidant contributor. These expand current understanding beyond enzymatic pathways.

Importantly, controls showed no significant changes, highlighting persistent OS in natural disease and the need for structured exercise. Notably, all HD patients had reduced UA postexercise. While HD clears excess UA, hyperuricemia remains prevalent and a CV risk [17]. UA has dual roles: antioxidant (serum levels correlate with total antioxidant capacity), but atherosclerosis‐promoting when abnormally metabolized. Thus, reduced UA postexercise presents a paradox—could lower UA coincide with diminished antioxidant defense? Deeper investigation into UA's role in MHD, especially with exercise‐induced metabolic adaptations, is needed.

Collectively, exercise potently modulates OS via direct (mitochondrial protection, ROS suppression) and indirect (antioxidant enzyme upregulation, metabolic modulation) mechanisms. Novel insights into UA dynamics emphasize targeted research to clarify its role in MHD pathophysiology.

### 4.2 Systemic Modulation of the Inflammatory Network by Exercise

Chronic microinflammation drives elevated infection susceptibility and cardiovascular morbidity in MHD [18]. A 6‐month supervised exercise intervention exerted systemic anti‐inflammatory effects: the exercise group showed significant reductions in pro‐inflammatory markers (IL‐6, PCT, CRP) and vascular injury biomarker (Hcy), with no changes in controls. These findings underscore exercise's capacity to regulate the inflammatory network through multi‐dimensional mechanisms.

First, reduced IL‐6—a key upstream activator of the NF‐κB pathway (regulating inflammatory mediators)—likely reflects exercise‐induced NF‐κB inhibition, dampening MHD‐related “cytokine storms.”

Second, synchronous decreases in PCT (bacterial infection/sepsis marker) and CRP (acute‐phase reactant) indicate exercise mitigates both acute and chronic low‐grade inflammation, critical drivers of long‐term MHD complications.

Third, reduced Hcy (endothelial dysfunction/vascular injury marker) may stem from exercise improving metabolic/vascular health. Exercise may counteract Hcy accumulation [linked to impaired endothelial nitric oxide synthase) activity and OS] by enhancing endothelial function (e.g., increased nitric oxide bioavailability) and modulating the methionine cycle, aligning with “exercise‐induced metabolic reprogramming” that restores redox balance/vascular homeostasis.

Collectively, exercise directly inhibits inflammatory factor release (e.g., IL‐6) and indirectly regulates markers (e.g., PCT, CRP, Hcy) via metabolic/vascular modulation. Targeting multiple inflammatory network nodes, exercise effectively reduces infection susceptibility and cardiovascular risk in MHD—bridging mechanisms and clinical relevance.

### 4.3 Synergistic Benefits of Exercise on Dialysis Adequacy and Body Composition

Dialysis adequacy (assessed by Kt/V) improved significantly in the exercise group versus controls postintervention (*p* < 0.05), likely due to enhanced systemic circulation, tissue perfusion, and endothelial function boosting urea clearance [19]. Though exercise did not significantly alter serum *β*₂‐microglobulin (a middle molecule toxin), controls showed mild accumulation over 6 months, suggesting exercise may mitigate its slow accumulation.

For body composition, controls experienced SMM decline, BMI/VFA/BF increase (aligning with sarcopenia/sarcopenic obesity risks). In contrast, the exercise group preserved SMM (less decline than controls) and reduced BMI/VFA/BF, driven by aerobic exercise (enhancing fat oxidation/insulin sensitivity) and resistance training (stimulating muscle protein synthesis). Both groups gained fat (reflecting MHD‐related metabolic dysregulation), but exercise mitigated this, lowering risks of metabolic syndrome, CVD, and functional decline.

Collectively, exercise optimizes dialysis adequacy (via clearance enhancement) and reshapes body composition (via muscle preservation/fat reduction), supporting its integration into routine MHD care for long‐term prognosis improvement.

### 4.4 Strengths and Limitations

This study has several key strengths: ① A supervised, personalized aerobic‐resistance training program with RPE‐adjusted intensity optimized adherence/safety and minimized intervention variability. ② Multidimensional assessments (OS, antioxidant systems, inflammation, body composition) provided a holistic view of exercise's multi‐target effects, avoiding fragmentation of single‐marker studies. ③ Strict protocols (fixed dialysis parameters, standardized medications, controlled diet) minimized confounding, ensuring observed changes were exercise‐attributable.

As a single‐center study, results may limit the generalizability to other populations due to variations in institutional practices (e.g., dialysis protocols, demographics). A 6‐month follow‐up also limits insights into long‐term sustainability (e.g., cardiovascular events, mortality) or delayed effects (e.g., *β*₂‐microglobulin accumulation). Additionally, the absence of a nondialysis CKD control group prevents distinguishing exercise‐specific effects from broader disease progression. Furthermore, due to spatial constraints in the dialysis unit, blinding of participants and personnel was not feasible, resulting in an open‐label design. This inherent limitation may have led to performance and detection biases, potentially affecting the internal validity of the findings. Although standardized protocols were implemented to minimize bias, the potential for bias cannot be entirely ruled out. Therefore, future multi‐center trials with extended follow‐up and nondialysis CKD controls and employing more rigorous blinding procedures are needed to strengthen generalizability and mechanistic insights.

In addition, the absence of functional outcome measures (e.g., 6‑min walking tests, chair‐stand, or handgrip strength). Although improvements in biochemical parameters and body composition were observed, the lack of functional data limits the clinical interpretability of these findings. Future studies should incorporate validated functional assessments alongside laboratory biomarkers. Integrating measures of aerobic capacity, muscle strength, and patient‐reported outcomes would provide a multidimensional assessment of treatment effects, clarify whether observed biochemical and compositional changes translate into tangible functional benefits, and help the development of personalized exercise interventions in nephrology care.

## 5 Conclusion

This study establishes that a 6‐month supervised, personalized exercise program‐combining aerobic and resistance training‐significantly attenuates OS, strengthens antioxidant defense systems, suppresses systemic inflammation, and optimizes dialysis adequacy and body composition in MHD patients. These findings provide robust, evidence‐based support for integrating structured exercise therapy into the clinical management of MHD populations. Future research should prioritize multicenter, large‐scale, and long‐term follow‐up trials to elucidate the sustained impacts of exercise on hard clinical endpoints (e.g., cardiovascular event rates, all‐cause mortality) and patient‐reported outcomes (e.g., health‐related quality of life). Such investigations will further solidify the role of exercise as a cornerstone of MHD care.

## Author Contributions

**Yuwei Chen:** conceptualization, methodology, project administration, writing – original draft, writing – review and editing, supervision. **Lu Yin:** methodology, data curation, formal analysis, writing – review and editing, visualization, validation. Conceptualization. **Xinzhou Zhang:** investigation, methodology, project administration, supervision, validation. **Xue Zheng:** data curation, formal analysis, and investigation. **Lijuan Lan:** supervision and visualization. **Liping Sun:** conceptualization, funding acquisition, investigation, visualization, supervision, writing – review and editing. All authors have read and approved the final version of the manuscript.

## Ethics Statement

The study was carried out after the protocol was approved by the Ethics Committee of Shenzhen People's Hospital (approval number LL‐KY‐ 2025147‐02).

## Conflicts of Interest

The authors declare no conflicts of interest.

## Transparency Statement

The lead author Liping Sun affirms that this manuscript is an honest, accurate, and transparent account of the study being reported; that no important aspects of the study have been omitted; and that any discrepancies from the study as planned (and, if relevant, registered) have been explained.

## Data Availability

All authors have read and approved the final version of the manuscript. The data that support the findings of this study are available on request from the corresponding author. The data are not publicly available due to privacy or ethical restrictions. The corresponding author Liping Sun has full access to all of the data in this study and takes complete responsibility for the integrity of the data and the accuracy of the data analysis.
